# Respirator masks protect health but impact performance: a review

**DOI:** 10.1186/s13036-016-0025-4

**Published:** 2016-02-09

**Authors:** Arthur T. Johnson

**Affiliations:** Fischell Department of Bioengineering, University of Maryland, College Park, MD 20742 USA

**Keywords:** Exercise, Respiration, Heat, Vision, Communications, Anxiety, Heart

## Abstract

Respiratory protective masks are used whenever it is too costly or impractical to remove airborne contamination from the atmosphere. Respirators are used in a wide range of occupations, form the military to medicine. Respirators have been found to interfere with many physiological and psychological aspects of task performance at levels from resting to maximum exertion. Many of these limitations have been investigated in order to determine quantitatively how much performance decrement can be expected from different levels of respirator properties. The entire system, including respirator and wearer interactions, must be considered when evaluating wearer performances. This information can help respirator designers to determine trade-offs or managers to plan to compensate for reduced productivity of wearers.

## Background

Respiratory protective masks (usually called respirators) are used whenever airborne contaminants are present and cannot be economically controlled by engineering means or administrative controls. Respirators come in many forms, including popular filtering facepiece respirators (FFRs), one-quarter, one-half, and full facepiece masks, and filtering air-purifying respirators (APR), air-supplied respirators, blower powered air-purifying respirators (PAPR), and self-contained breathing apparatus (SCBA). They are used by personnel in homes, industry, agriculture, mines, emergency first responses, medicine, and the military wherever airborne contamination is a possible threat [56]. The threats may be from gases, vapors, dusts, and particulates of various sizes, including aerosols [19].

Although the protective mechanisms of respirators are largely physical and sometimes chemical, wearing respirators come with a host of physiological and psychological burdens [15]. These can interfere with task performances and reduce work efficiency. These burdens can even be severe enough to cause life-threatening conditions if not ameliorated. Quantitative assessments of these burdens have been made so that respirator design trade-offs, wearer usages, and regulations can accommodate the needs of the wearer [7, 36, 55].

Understanding possible physiological and psychological effects of respirator wear requires a thorough understanding of the wearer and possible respirator effects [23]. Respirators may appear to be rather simple, but they can interfere with [36, 55]:

1. respiration
2. thermal equilibrium
3. vision
4. communication
5. feelings of well-being
6. personal procedures such as eating and sneezing
7. other equipment

There are two basic principles relevant to respirator use:

1. Work cannot usually be performed as long or as hard while wearing a respirator compared to when respirators are not worn. Wearing protective clothing plus respirators makes this situation even worse. Either more time must be allowed for a particular task or more workers must be assigned to the same task.
2. There is a great deal of wearer variability. Some wearers can tolerate respirator high inspiratory or expiratory resistance or pressure levels, while others cannot. Some wearers are much more anxious about wearing respirators than others. Some wearers can tolerate hot, humid conditions inside respirators, whereas others cannot. Because of this variability, each wearer must be treated as an individual.

## Physiological reponses to work activity

**Fig. 1:**
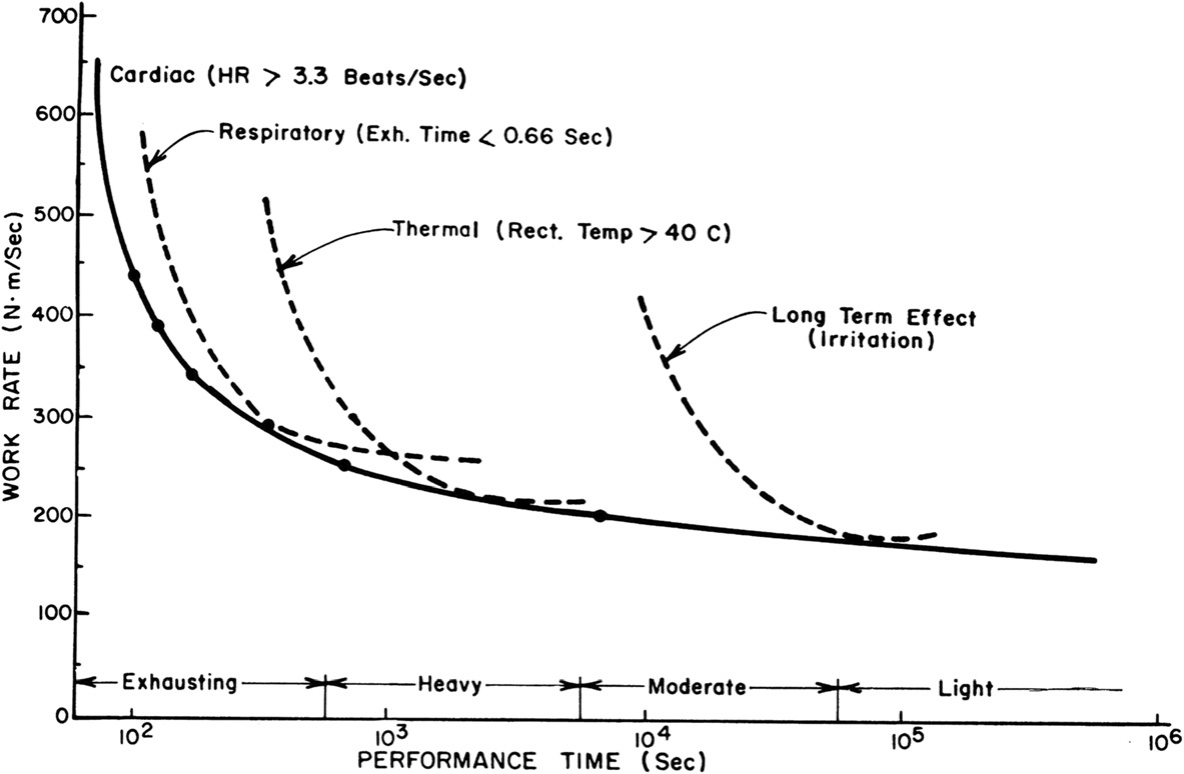
Schematic representation of performance time while exercising wearing a protective mask

A brief discussion of ergonomics and work physiology is necessary to understand heavy exertion while wearing respirators and protective clothing [12, 17, 27]:

1. Work/performance time tradeoff
  Very hard work cannot be performed for as long a time as work of lesser intensity (Fig. 1). This is true even when unencumbered by protective equipment. In the Figure, it can be seen that, for different activity levels, there are corresponding physiological limitations consisting of a cardiovascular limitation for very intense work, respiratory limitation for intense work, thermal limitation for moderate work [21], and what is generally called irritation limits for low-level activity [13, 17, 27]. Protective masks and clothing generally shorten the time that a particular activity level can be sustained.
2. Physiological adjustments
  The human body is attuned to performing physical labor. What follows the start of muscular activity is a coordinated series of adjustments [12] involving all parts of the body, including the heart, blood vessels, the lungs, digestive system, nervous system, and the kidneys. The ones with most direct bearing on exercise adjustments are described below.

## Metabolism

Muscular movement requires energy [17]. This energy comes from an energy storage molecule called ATP (adenosine triphosphate). When the supply of ATP is exhausted, muscle activity ceases. It is important, therefore, to replenish the ATP supply as quickly as possible in order to maintain muscular work. There is also another energy-rich compound in the muscles called creatine phosphate that can act to replenish the ATP supply extremely quickly. When the muscle starts working there is enough ATP in the muscles to sustain the work for 0.5 sec. There is enough creatine phosphate present to keep the muscle working for up to 2 min. After that, other energy- transforming mechanisms are necessary to replenish the ATP supply.

**Fig. 2:**
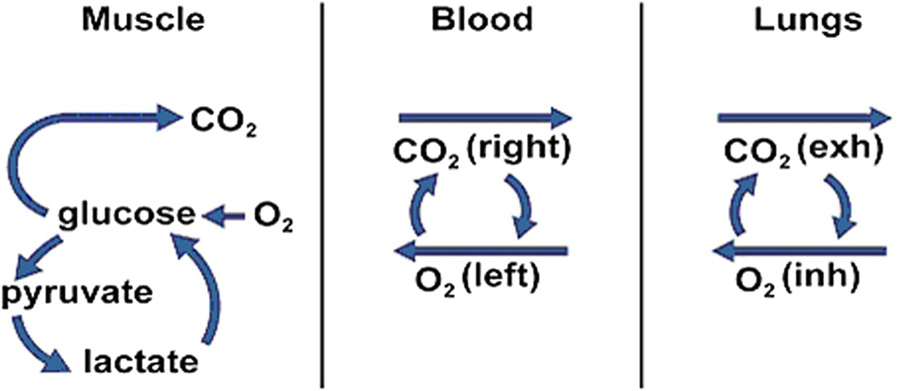
Oxygen delivery to the muscles is a multistep process, beginning with gas exchange in the lungs (right), being transported in the blood (middle), and finally being used in the muscles (left)

This other energy comes from stores of glucose in the blood, glycogen (an animal form of starch) in the muscles and liver, fats in the form of triglycerides in fat tissue, and body proteins. In order to extract the energy from these compounds, they must be respired at the cellular level, and there are two kinds of cellular glucose respiration: anaerobic and aerobic. The difference between the two is that aerobic respiration requires oxygen and anaerobic respiration does not. Oxygen delivery to the muscles begins in the lungs, continues in the blood, and is finally delivered to the muscles (Fig. 2). If enough oxygen can be delivered to the tissues, then aerobic respiration can keep up with the energy demands of the muscles. However, there are limits to the rate that oxygen can be supplied, called the maximum oxygen uptake, and, once the maximum oxygen uptake is reached, additional muscular energy must come from anaerobic respiration.

Very heavy exertion requires at least some anaerobic respiration because oxygen demand exceeds the maximum oxygen uptake. This is called the anaerobic threshold. Anaerobic respiration yields 18 times fewer ATP molecules [18] than does aerobic respiration, and so is not nearly as efficient. However, it does allow movement to continue, at least for a while.

One of the end products of aerobic respiration is carbon dioxide, which can be removed during exhalation. Carbon dioxide levels in the exhaled breath rarely reach more than 4–5 % even at the extreme, but, if it did climb much higher, carbon dioxide can cause disorientation, confusion, and even death [1, 17].

The main end product of anaerobic respiration is lactic acid that is released from the muscles into the blood [17]. There are buffering mechanisms in the body that tolerate lactic acid additions, but these mechanisms have limited capacity. Once this capacity is reached, there is no other source of energy for the muscles and all muscular activity must cease. This capacity to tolerate lactate is called the maximum oxygen debt because all the lactic acid must be reformulated into pyruvate at the end of exercise, and this requires oxygen.

Buffering the blood against lactic acid formation during anaerobic respiration produces extra carbon dioxide that can be exhaled. This extra carbon dioxide acts as a respiratory stimulant that leads to hyperventilation, or harder and deeper breathing.

All these processes proceed each time a person moves actively. They are much more efficient for younger people than for older people. Maximum oxygen uptake for 20 year olds is about 2.5 l per minute, but declines nearly linearly to about 1.7 l per minute at age 65 [17]. Well-trained individuals can have maximum oxygen uptakes up to twice these values. In addition, the maximum oxygen debt that can be incurred by an individual declines with age and is also affected by training [12].

Metabolic responses during exercise, and especially during emergencies, are modified by the release of the adrenal hormones adrenalin (epinephrine) and cortisol. These hormones increase metabolic rate, increase the rate and force of heart contractions, enhance the availability of blood glucose, reroute blood from the gut to the muscles, and mobilize the nervous system. The combined actions of these hormones can affect physical, emotional, and cognitive functions.

Muscular strength declines with age, making task performance less efficient when more muscles must be recruited to perform a task. Muscular power can be restored relatively rapidly with strength training.

Drugs and medicines can also affect body metabolism, as can illness. Products of cigarette smoking and caffeine also affect metabolic rate [65].

## Cardiovascular adjustments

The heart adjusts to the physical demands of exertion by increasing its cardiac output, or the volume rate of blood flow through the arteries, capillaries, and veins. This is done to increase the rate of glucose and oxygen supplied to, and removal of lactate and carbon dioxide from, the muscles. The heart rate increases nearly linearly with work rate, beginning to increase nearly as soon as work rate increases. This is due to kinesthetic neural sensors in the muscles and joints that signal the fact that increased oxygen demand is on its way (feedforward control), despite the fact that there is as yet no reduction in blood oxygen concentration or rise in carbon dioxide concentration. Once the concentrations of these gases change, then control of heart response is determined by chemical sensors in the aorta, in the carotid arteries in the neck, and in the brain [17].

The stroke volume of the heart, or the volume of blood pumped for each heart beat, increases initially at the start of exercise, but soon reaches its maximum level. Thereafter, increases in cardiac output are determined only by heart rate. Cardiac output at rest is about 5 or 6 l per minute; cardiac output can rise to 25 l per minute during strenuous activity. Blood volume in a somewhat smallish 150 lb (70 kg) person is about 5.6 l. Hence, it takes about 1 min at rest and 12 s during exercise for blood to make the loop of the whole circulatory system.

Larger people generally have larger hearts and larger stroke volumes [18]. Well-trained individuals have lower resting heart rates and higher resting stroke volumes. Older individuals can have somewhat lower cardiac efficiencies than younger individuals [17].

If body temperature rises due to overheating, then blood is shunted to body surface vessels and there is a secondary rise in heart rate, which puts additional stress on the heart. The water from sweat is derived from the blood plasma, causing the blood to thicken somewhat during prolonged exercise [17]. This also increases stress on the heart, but is alleviated by drinking sufficient amounts of liquid, some of which can be drunk before or during work, if available.

Cardiovascular adjustments also include shunting the blood from maintenance activities, such as digestion and kidney function, to working muscles where it is needed. Much of the blood in the circulatory system at rest is located in the leg veins; during exercise, most of the blood is shifted to the arteries. These changes occur very quickly after activity begins. Release of the hormones epinephrine and cortisol during psychological stress speeds the heart and constricts some blood vessels to shunt blood to the arms and legs.

Oxygen delivery to the working muscles can be limited by the maximum cardiac output, given as the maximum heart rate times the maximum stroke volume [17]. Once this maximum has been reached, metabolism continues anaerobically. Depending on the muscles being used and the vascular structure serving those muscles, there may be local regions of anaerobic metabolism occurring while the muscles as a whole are still aerobic.

### Cardiovascular limits

There is a maximum heart rate that can be achieved by an individual. This is age dependent, generally being able to be predicted as 220-(age of the individual). Younger people therefore have higher maximum heart rates [17]. Once this maximum heart rate is reached, cardiac output no longer increases, and oxygen delivery to the muscles becomes static. Anaerobic metabolism is incurred, terminating when the maximum oxygen debt in reached. Cardiovascular-limited exercise normally terminates in 2 to 4 min.

### Respirator effects

Data from multiple studies have shown that the use of respirators by themselves have no effect on heart rates of the wearers [46]. From this, it appears that respirators do not impose additional stress on the heart. However, for respirators and protective clothing with significant weight, the additional weight can impose an ergonomic burden that translates into cardiac stress. This additional weight acts equivalently to body weight as long as it is carried close to the body. Each kilogram (2.2 lb) of extra weight can be expected to reduce the work performance time by 2.5 min if walking at a high rate of speed [49].

If extra weight is carried awkwardly away from the body, then the energetic penalty can be an additional 50–60 % of the energetic cost of carrying the load next to the body [17]. Extra heavy loads add, as well, to the nonproportional energy cost of carrying them. Loads carried by the hands are less burdensome than loads carried on the feet. Heavy protective clothing carries with it a higher energy penalty than can be accounted for by its weight alone. Apparently, bulk and friction of the clothes is also an important factor.

Translating the energy requirement of wearing protective clothing and carrying (or dragging) extra weight into cardiac burden is not a straightforward procedure. A lot depends on whether climbing up stairs, down stairs, or walking on the level; the texture and compositions of walking surfaces; the speed of movement; and the body temperature of the wearer. Under relatively easy walking conditions, the increase in heart rate while carrying an extra 60 lb (27 kg) of weight is a heart rate increase of 10 % of the maximum [17].

## Respiration

Respiration also increases as exercise progresses, but respiratory responses lag activity level changes by about 45 s [17]. There are many respiratory responses that occur: the respiration rate increases [5, 41, 42], the tidal volume (or the amount of air breathed during each breath) increases up to a maximum amount, the respiratory waveform changes [40], there are adjustments to the airways, and lung volumes change [14]. Many of these changes appear to be stimulated by carbon dioxide concentration of the blood, but initial respiratory adjustments occur too quickly for that to be the only determinant; kinesthetic sensors may also be important for initial respiratory adjustments [64].

Respiration is a multistep process, whereby air is breathed in, travels through the airways, reaches the alveoli (the sacs at the end of the lung where gas exchange takes place), diffuses across the alveolar membrane, dissolves in the blood, and is absorbed by the hemoglobin in the red blood cells. Carbon dioxide diffuses rapidly into the blood, so the concentration of carbon dioxide in the alveoli and the blood are always equal, even during the most intense activity level. Oxygen, on the other hand diffuses more slowly than carbon dioxide, so its concentration in the blood is lower than in alveolar air during inhalation [17]. Diffusion rates of both gases change somewhat with activity level, with those for men being somewhat higher than those for women.

Inhaled air is oxygen rich and carbon dioxide poor. Exhaled air is oxygen poor and carbon dioxide rich. Because air flow in the airways is bidirectional, the first air that reaches the alveoli is the same as the last air that was exhaled during the previous exhalation. This is an indication of the dead volume of the lung, or that volume that stores carbon dioxide from the previous breath. Dead volume for average adults is about 180 ml, but dead volume of respirators can add to the effective dead volume of the respiratory system and affect performance [52].

**Fig. 3:**
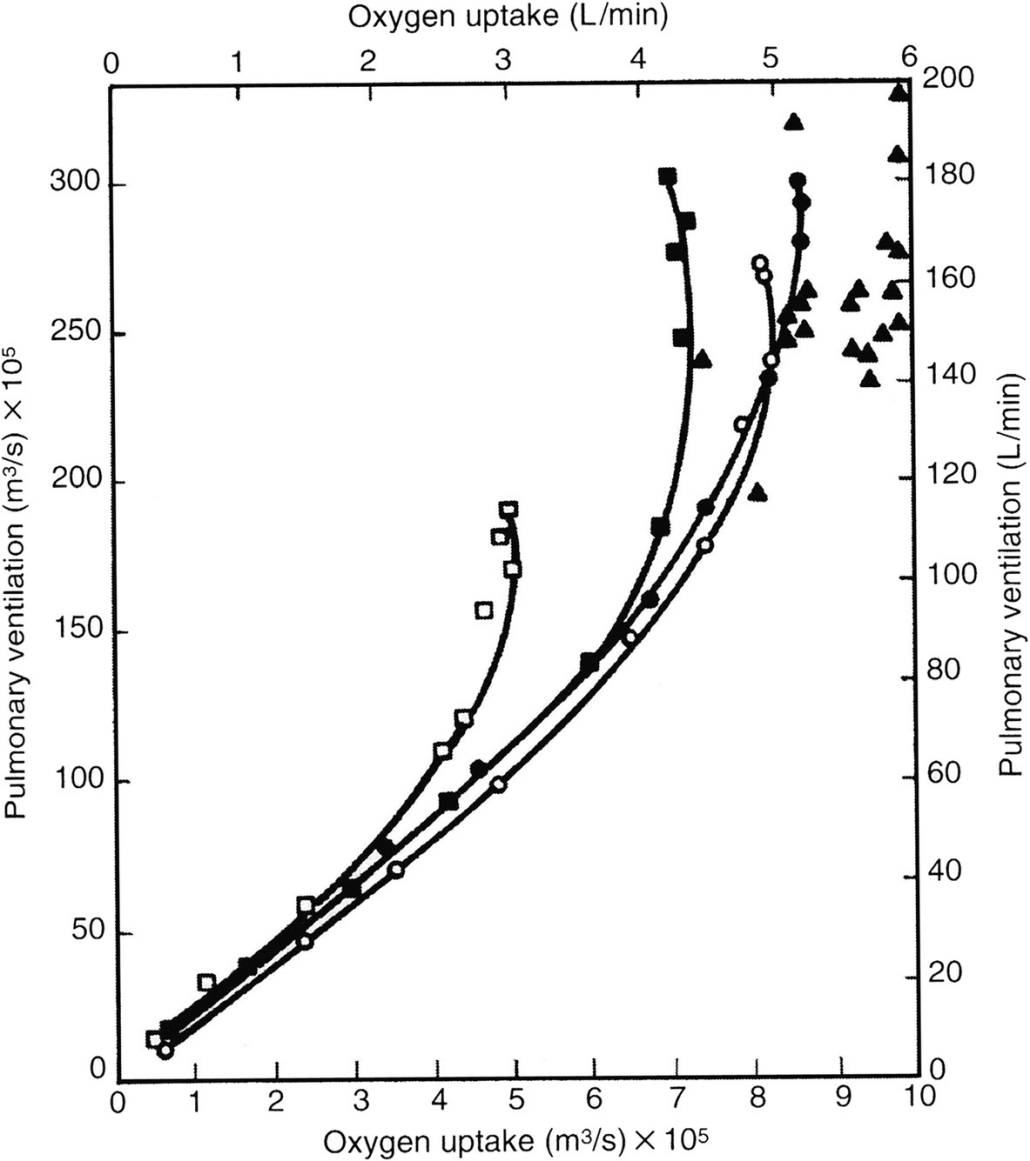
Pulmonary ventilation increases linearly with oxygen uptake until the anaerobic threshold, and then dramatically greater once stimulated by additional carbon dioxide

Carbon dioxide is a very powerful respiratory stimulant. Increasing the concentration of inhaled carbon dioxide increases lung ventilation much more than does oxygen deficiency. Metabolically-produced carbon dioxide is even more effective than inhaled carbon dioxide at stimulating respiration. This is critical for additions of external dead volume, which transforms exhaled metabolic carbon dioxide into carbon dioxide inhaled during the next breath. Once the anaerobic threshold is reached, blood buffering makes it appear that metabolic carbon dioxide increases, and respiration is stimulated so much that lung ventilation increases dramatically as work rate intensifies (Fig. 3).

Working muscles change their efficiencies over time as they heat and tire. Additional oxygen demands of muscles that have been worked for several minutes increases the need for the respiratory system to respond. This leads to a secondary rise in lung ventilation that continues well into the exercise duration.

Moving the chest wall, lung tissue, and air in the airways requires energy. This energy is equivalent to about 1–2 % of the total body oxygen consumption at rest, but increases during intense activity to 8–10 %. For people with obstructive pulmonary disease, the percentage at rest can be 18–20 %. These people cannot perform strenuous exercise. Adding external resistance or dead volume from a respirator (APR), or external pressure (SCBA or tight-fitting PAPR), increases the amount of work that must be supplied to breathe. Oxygen to supply the needs of the respiratory system cannot be used to supply the working muscles, so respiratory demands can definitely limit the rate of work that can be expected of a wearer [13].

The work of respiration is supplied by the respiratory muscles. These include the diaphragm, the intercostals, and the abdominals. Inhalation is caused mainly due to the straightening of the diaphragm in the chest. Exhalation at rest for those without obstructive or restrictive pulmonary diseases (normal, healthy individuals) is passive; that is, the force to propel the air to leave the lung comes from the elasticity of the stretched lung. Exhalation during exercise needs to happen a lot faster than during rest, so becomes active when the abdominal muscles push air out of the lung. Due to this difference, it is much easier and comfortable to breathe against PAPR or SCBA positive pressure during exertion than during rest.

The airways are reactive, and change during exercise. They can constrict somewhat to reduce dead volume, and thus lower wasted breathing effort, but, as they constrict, they resist air flow and increase the work of breathing [37], so there is a dynamic level of airway tone that is achieved. These same airways may constrict to protect against respiratory irritants reaching the lung, and cause the same symptoms as a severe asthma attack.

### Respiratory limits

Respiration does not usually limit work performances of healthy individuals, but respiration can limit work time when respirators are worn [44, 51]. The most important function of the respiratory system is the removal of carbon dioxide from the body. Adjustments during exercise increase depth and rate of breathing in order to expel this gaseous end-product of aerobic metabolism. Exercise exhalation becomes actively supported by the abdominal muscles, spewing carbon dioxide at faster rates as exercise intensifies. At some point, the rate at which air can be exhaled becomes limited by the distensible airways in the respiratory system. Any further increase in abdominal pressure cannot increase expiratory flow rate. Thus, for normal individuals, there is a limitation when exhalation time decreases to one-half second or so [22, 24]. Carbon dioxide cannot be expelled any faster than this minimum exhalation time allows. Additionally, some people suffer from respiratory impairments that limit maximum pressures that can be generated by the respiratory muscles when they breathe through external resistances or against external pressures [59]. Respiratory-limited work usually lasts 5–20 min.

### Respirator effects

Air-purifying respirators (APRs) have inspiratory resistances dominated by filter resistances, with a typical value of 3.5 cm of water-seconds per liter (or 50 mm H_2_O at 85 L/min flow rate). Exhalation resistances of the exhalation valves may be somewhat less than 1.5 cm H_2_O-sec/L. Powered air-purifying respirators (PAPRs) may have much lower inhalation resistance but the same exhalation resistance. Self-contained breathing apparatus (SCBA) may have zero or negative equivalent resistance, but very high pressures to exhale against. Although exhaling against high pressures is uncomfortable at rest, when respiration usually includes passive exhalation, high-exhalation pressures can be tolerated better during exercise when the respiratory muscles for exhalation contract actively. Previous work seems to indicate that inspiratory and expiratory resistance effects are equivalent [3, 4, 50], although testing using very high expiratory resistance resulted in severely degraded performance [31]. No thresholds for respirator resistance effects has ever been detected, so FFRs with very low resistance values would still be expected to have some effect.

The effects of APR inspiratory resistance on performance are felt most at very intense exercise (80–85 % maximum oxygen consumption). Performance time decreases linearly with increased inspiratory resistance at this exercise intensity [50]. A resistance level of 3.5 cm H_2_O-sec/L is expected to result in a 30 % performance decrement. Because of this, one might expect performance with PAPR to be better than with air-purifying respirators, but this has yet to be definitively answered, and the extra weight of the blower and tubing may counteract at least some of the advantage of lower resistance [51, 63].

Extra inspiratory resistance [38] promotes hypoventilation [2–4, 6, 16, 39, 50, 60] of the wearer (lower volumes of air breathed and smaller amounts of oxygen used). This can result in an earlier transition from aerobic (using oxygen) to anaerobic (no oxygen needed) respiration [10, 32], and faster progress toward the maximum tolerance for exercise (maximum oxygen debt).

Facepiece dead volume accumulates exhaled carbon dioxide in the voids between the respirator and the face and returns it to the respiratory system during the next inspiration. This carbon dioxide then acts as a respiratory stimulant. Because carbon dioxide is a psychoactive gas, dead volume may also produce discomfort and a performance decrement at low-intensity work. A typical value for full-facepiece APR respirator dead volume is 350 mL. Such a dead volume is expected to reduce performance time by 19 % at a work rate of 80 to 85 % of maximum oxygen uptake [52].

Intense exercise above the anaerobic threshold uses more air than does moderate exercise, and because very intense exercise metabolism has a higher anaerobic component than does moderate exercise, the air that is used is not consumed as efficiently as it is at lower intensity [43]. The net result is that SCBA tank air depletes much more rapidly at high work rates than at moderate work rates.

## Thermal responses

The large skeletal muscles are only about 20 % efficient [20]. Of the energy supplied to the muscles, approximately 80 % ends up as heat. Thus, heat loss mechanisms are necessary to maintain thermal equilibrium of the human body.

These mechanisms include vascular adjustments, sweating, and voluntary responses. Voluntary responses include moving to cooler locales, stretching out to lose more heat, drinking cool liquids, or removing heavy clothing. These responses may generally be unavailable to workers or emergency responders who need to protect against threats of unknown type in uncontrolled environments.

There is a thermal mass to the body that requires some time for heat to build up and cause dangerous body temperatures. There is a normal 6–10 min of activity that can occur before deep body temperature rises significantly [17]. Skin temperature probably increases during this time. If sufficient heat cannot be lost to the environment, then body temperature will continue to rise until it reaches dangerous levels. A core body temperature of 104 ° F (40 ° C) is expected to give a 50 % casualty rate [11]. This condition is characterized by disorientation, convulsions, loss of body temperature control, and death.

Heat can be lost from the body by convection (usually, air movement), radiation (as to a cold clear sky), or evaporation. Convection and radiation heat loss depends on the difference in temperature between the surface losing heat and the surrounding fluid (usually air, but, in a pool, water). Thus, one adjustment the body makes during thermal stress is to warm the skin surface. It does this by shunting blood from deep veins into surface veins. This is why veins on the surface of the hands seem to stand out more in hot weather than in the cold. There is also a small, but significant, amount of convective heat loss from the respiratory system as air is breathed.

Evaporating water absorbs a large amount of heat, thus making sweating effective as a heat loss mechanism. Sweating heat loss on the surface of the skin is nearly 100 % effective for losing heat. Sweating through clothing cools the clothing surface where the evaporation actually takes place, and only partially cools the skin. Sweat that drops from the skin is completely ineffective for heat removal. The amount of sweating depends on the cooling necessary, and different parts of the skin are recruited at different times to produce sweat. When fully recruited, the maximum cooling that can be obtained from sweating is equivalent to nearly 12 times the body heat production at rest (or 11.4 mets).

Women have higher percent body fat than do men. They use this body fat as insulation between their body cores and the outside environment. To lose heat, therefore, women depend more on vascular adjustments that do men. Men sweat more than women and lose a larger fraction of their heat in that way. Acclimation to hot environments can improve sweating efficiency by increasing both the rate of response and amount of sweat produced.

Some workers may not need to wear protective clothing with their respirators. However, covering up the entire body, and possibly moving into a hot environment eliminates nearly all possibility of heat loss natural to the human body. Other means must be provided, such as supply of cool air from an SCBA or PAPR, or body temperatures must be closely monitored. An alternative is to limit heat exposure time and to provide adequate rest cycles.

Some work may be required in very cold temperatures [66]. At the beginning, cold temperatures may limit movement and dexterity. However, heat produced during activity and the extra insulation afforded by protective clothing and respirators soon overcome cold temperature effects on the body. Surface blood vessels in the head do not constrict in the cold, as do similar blood vessels in other parts of the body. Hence, nearly half of the body’s heat loss in the cold can come from the uncovered head. Covering the head and face with protective equipment helps to insulate against this large amount of heat loss.

### Thermal limits

The most important work limitation associated with heat is deep body temperature. It must be prevented from reaching 40 ° C. A conservative limit for adults might be 39.2 ° C (102.5 ° F). Beyond this, thermal discomfort becomes overwhelming and death could ensue. Muscular efficiency is reduced at high temperatures and judgment ability becomes impaired. Thus, the overheated individual cannot be expected to recognize his or her own dangerous situation [62].

Because of the thermal capacity of the body to store heat, it takes a while before body temperature rises to the point where it can become limiting. Heat-limited work usually occurs in the 10 min to 2 h time range.

### Respirator effects

Use of respirators in nontemperate conditions can lead to special problems. [35] Cold conditions can cause fogging of full facepiece respirators, which leads to severe dissatisfaction with respirator use. Nose cups inside the facepiece are designed to eliminate fogging, but are not always effective. Fog-proof lenses are available on some models. Fog-proofing solutions that can be applied to the face shield are also available. Cold can also cause valve sticking and stiffen the rubber facepiece material to the point that it prevents a good facial seal. Cold rubber has a higher thermal conductivity than does still air, so in still, cold air the face may be cooled by the respirator. In a cold wind, however, the facepiece may add a small amount of insulation to the face.

Use of respirators in hot conditions leads to several difficulties. Discomfort has been related to facial temperatures inside the facepiece. Facial skin temperatures are more important for comfort than skin temperatures in other parts of the body. PAPR blowers send filtered air over the face that evaporates sweat and cools the face [51]. SCBA air expands and cools when released from the cylinder; this cool air can also help to alleviate facial discomfort. Some SCBA have coolant packs used to further cool supplied air before it reaches the facepiece. APRs, however, have been found to be uncomfortable in the heat because they do not supply cool air.

At moderate work rates (50 to 70 % of maximum oxygen uptake, or maximum exercise capacity), respirators impede the loss of heat from the face and can result in hyperthermia occurring sooner than it otherwise would. This is not usually a problem except when the rest of the body is sealed in protective clothing. With no easy means to lose heat, the body can overheat, especially in hot and active conditions.

Sweat produced inside the facepiece can accumulate and cause discomfort, interfere with breathing, and cause exhalation valve sticking [48]. Accumulated sweat can cause a respirator face piece to slip on the face and promote leakage.

**Fig. 4:**
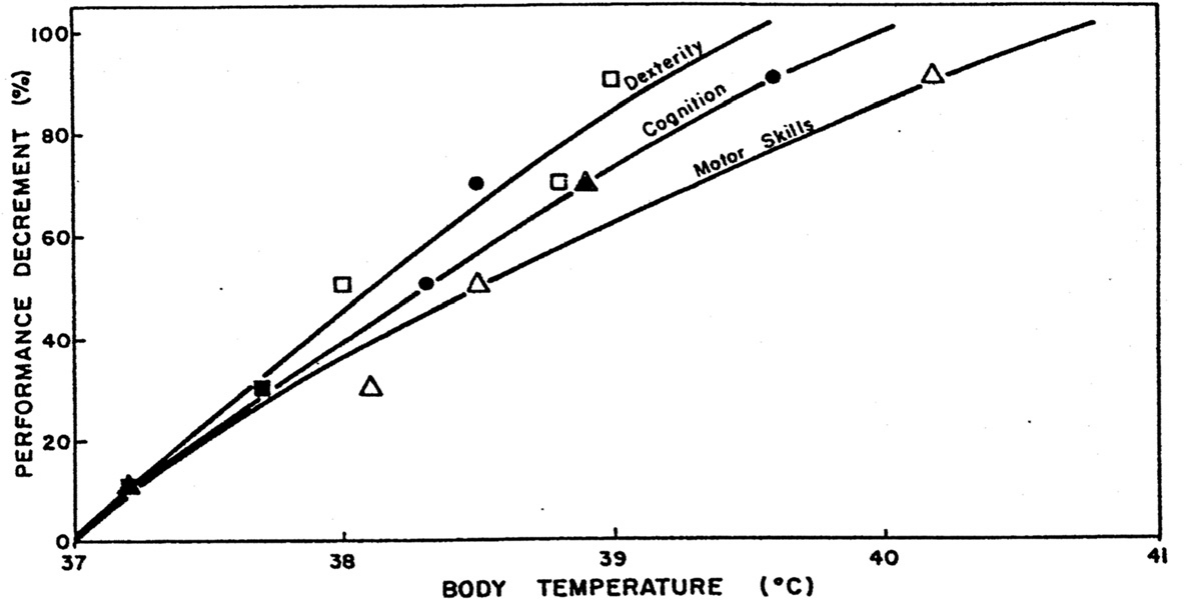
Effect of body temperature on dexterity, cognition, and motor skills

There is also an effect of heat on the ability to recognize dangers, make coordinated movements, and perform manual tasks [35]. As deep body temperature increases, dexterity, cognition, and motor skills degrade significantly (Fig. 4). One of the most dangerous effects of overheating is disorientation, and not being able to recognize the direction to safety in the event of extreme danger. This inability to recognize safe passage has contributed to past deaths. Respirators can contribute to this disorientation by adding other sensory burdens (especially vision and hearing) to the wearer in this environment.

## Prolonged activity

Some wearers will be assigned tasks that are physically not very intense. These people will have no trouble with maximum oxygen debt, maximum oxygen uptake, or (most likely, unless the ambient temperature is extremely warm) excessive body temperature. Different challenges confront these wearers. First of all, discomfort is felt more strongly when attention is not directed elsewhere. There can be a considerable amount of discomfort associated with wearing respirators, gloves, boots, and protective suits. Those individuals prone to anxious feelings may have their anxieties made worse during periods of inactivity. Anxieties are the most important threat to protective equipment wear, and extremely anxious people should not be asked to wear respirators, if possible. Studies have shown that anxiety level is a very reliable indicator of difficulty encountered while wearing a respirator. Extremely anxious individuals do not perform for as long or at the same work rate as low-anxiety wearers [28, 61].

For those who can tolerate the discomfort and claustrophobic feelings when wearing respirators, there will nonetheless be physical effects of prolonged wear [54, 57]. Many respirators require a tight face seal in order to assure adequate protection. The site of the face seal may produce rashes and edema in surrounding skin areas. These will disappear with time once the equipment is removed.

Vision can be important at low work rates. There may be tasks to be performed that require a broad visual field or fine discrimination among various lights, switches, or objects. Respirators interfere with vision in various ways, but visual acuity at low work rates can be compromised by lens fogging, dust or films on the lenses, or wearing of improper corrective lenses. Sweating people wearing respirators in cold drafts can easily incur moisture condensation inside the facepiece. Dusts and precipitates that are of no respiratory consequence to the wearer can obscure vision if not able to be wiped from the lenses.

### Prolonged activity limits

Physiological limits to long term exercise deal with limitations on blood glucose levels and muscle glycogen stores. Dehydration or electrolyte depletion may occur [17]. These are difficult to quantify for any individual, but frequent eating and drinking can deter them from happening [30].

Psychological effects are also important. Feelings of fatigue are common, as are feelings of anxiety and discontent [54, 57].

### Respirator effects: vision

Sharp vision is important for some of the tasks required during an emergency. There is a natural tunneling of vision that occurs during intense exertion: attention is focused on objects straight ahead. Consequently, degradation of vision due to respirator use during high exertion has little effect on the ability to complete the required task. Under normal conditions, this might be advantageous to task performance. In a situation where dangers can come flying from all directions, there may be difficulty recognizing peripheral threats.

Vision is extremely important for performing some tasks, such as computer work, console monitoring, and reading [9, 29, 33, 34]. There are many aspects of vision, including visual acuity, peripheral vision, and color detection, and some or all of these may be needed. Respirators should be selected to accommodate requirements for peripheral vision, acuity, and color recognition.

Workers requiring corrective lenses while wearing full facepiece respirators must not wear spectacles with temple bars or straps that come between the sealing surface of the respirator and the face. Instead, special corrective lens mounting kits may be used with full facepiece respirators. These may not be entirely satisfactory for some wearers. Those who can wear contact lenses can usually do so while wearing a respirator mask. As long as the insides of full facepiece respirators are kept clean, dust particles will not be present to cause difficulties with contact lenses.

**Fig. 5:**
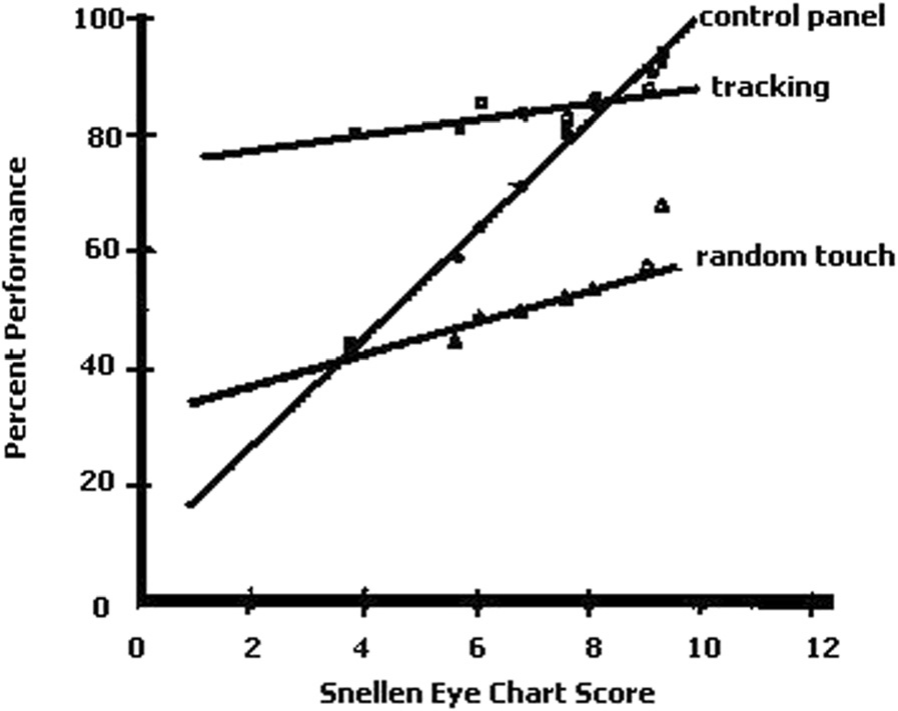
Performance for several tasks as visual acuity varies while wearing respirators. The Snellen eye chart denotes better vision for higher line numbers. Control panel recognition and performance ability is particularly sensitive to visual acuity

Dust, mist, smoke, condensation, or water flowing down over the facepiece lenses can degrade visual acuity during an emergency. Under such conditions, task performance can be expected to be seriously degraded (Fig. 5), and extra training under these conditions might be warranted [29]. Disorientation in a low-visibility environment is common, and may make it difficult to know how to move or which direction is the safest to go.

Although visual acuity has little to no effect on performance of intense physical activity [33], wearing a full facepiece respirator while walking, running, or driving can erode visual acuity somewhat, probably due to the pull of the shaking facepiece on the face. Recognition of objects or signs while wearing a respirator and walking or driving cannot be expected to happen as quickly as without a respirator.

### Respirator effects: communications

Full facepiece respirators can interfere with visual cues during speaking and listening. It thus becomes more difficult not only to recognize what is said, but also who is saying it. Distance and intelligibility are interrelated; longer distances between communicating individuals result in less intelligibility. Speakers and listeners should talk in sentences where the message can be conveyed by context as well as by word recognition. Sentence context allows speakers and listeners to be separated by 10 times the distance compared to communicating by single words. Simple words and phrases are unable to be understood 27 % of the time at distances as close as 2 ft [8].

When telephones or radios are used for long-distance communication, expect a 10 % error rate in recognition of words and a 50 % increase in the time required to recognize the words [45, 47, 53]. Because standard telephone and radio equipment dimensions are not entirely compatible with respirator facepieces, protocols should be established to let the user know when to move the earpiece from the ear and to move the mouthpiece in front of the speech diaphragm. Training in the use of these protocols is essential [58].

Special communication equipment is available from some manufacturers and some respirators have speech diaphragms or are made of materials that enhance speech transmission.

If workers are close enough to be able to see each other, a lot of communication can take place with hand signals. There are some generally-accepted hand signals that denote easily-understood simple messages (examples of these are thumbs up for agreement, a finger across the throat for danger, an upright palm to indicate “stop”, and pointing to indicate direction). These will be harder to see in a dusty or smoky environment and with gloves on, so there is a distance penalty even with hand signals.

One of the most difficult impediments to clear communication is accented speech. If speech cannot be clearly understood without a respirator, it will be nearly impossible with a respirator. Hand signals may serve to overcome speech understanding, but different cultures may also have different interpretations of hand signals.

### Respirator effects: bulk

Respirators can interfere with worker activities because of their bulk or weight. Use of respirators in tight places is difficult and can temporarily disrupt facial seals when bumping against other objects. Respirators may interfere with sighting equipment or with other measuring devices. Contrarily, the impact resistance of the lenses of many full face piece respirators can be a positive attribute in situations where objects or debris may possibly fall to the face.

Protective clothing can be bulky and heavy, and can impede worker progress. Small spaces must be larger for a protected worker to fit into. Gloves make fine hand or finger movements nearly impossible.

### Respirator effects: personal procedures

The facial area inside a respirator is usually not accessible from the outside unless the face seal is broken. Thus, eating, drinking, scratching one’s face, blowing one’s nose, or rubbing an eye are not possible while wearing full facepiece respirators. One exception to this is certain respirators that have a drinking tube incorporated into their designs.

As long as periodic breaks are allowed, respirators should not add to the fatigue that accompanies long term work [30]. Food or drink can be ingested during those breaks, and energy levels maintained. While it is unlikely that responders would be needed to work for hours at a time without breaks, if such were the case, then blood glucose could fall to dangerously low levels (hypoglycemia), and work could not continue efficiently.

The inaccessibility of the face may generate considerable tension in the mind of the wearer, especially if the reason to access the face is due to some particularly sensitive need. Dust or dryness in the eyes of contact lens wearers, runny noses, or unbearable pressure to parts of the face can be particularly distressing. If the situation does not allow the wearer to leave the hazardous environment to take care of the problem, then considerable anxiety may develop.

## Work/rest cycles

As given in Fig. 1, more intense work cannot be sustained as long a time as can less intense work. If workers are expected to work very hard for a while, they must also be in a position to rest or, at least, slow down for a while. This can be a problem if the worker cannot control the rate of work, because anaerobic work continued for too long can result in the maximum oxygen debt being reached. Then the worker would not be able to work any more until he or she recovers sufficiently.

The amount of time that a person can be expected to work is related to the fraction of the maximum oxygen uptake represented by the task being performed [17, 26]. Thus, performance time involves the size of the individual as well as age, sex, and physical conditioning. In general, men have higher maximum oxygen uptakes than women, but they have larger sized bodies that use more oxygen to move around. Older people have lower maximum oxygen uptakes than younger people. Wearers in better physical condition have higher maximum oxygen uptakes, and, additionally, are able to perform tasks with lower oxygen use than are less physically-able wearers.

Work performance times can range from forever at rest, to 4 h walking at 3 miles per hour, to 23 min for cross-country running, to 10 min climbing stairs. These are typical times for an unencumbered 40 year old man [17]. The addition of extra protective equipment can reduce these times to one-half or less of the values given, depending on the types of equipment worn.

Rest times are also dependent on the intensity of the task and the maximum oxygen uptake of the individual [17]. In general, the more intense the work, the longer will be the recovery time, but the relationship is nonlinear. A task that can be performed for an hour requires at least a 10 min rest period. More intense tasks (with shorter performance times) require longer rest times.

## Conclusion

Physical exertion involves the entire body in a coordinated fashion. Adjustments made during work or exercise can be profound, but the limitations of exercise can be modified or overcome by training and proper selection of equipment. Familiarity with the physiological adjustments that occur can lead to enhanced effectiveness and larger return on investment for both manpower and equipment [25]. As long as humans are involved in performing physical or mental work, accommodation must be made for the adjustments that characterize their physical abilities. Training is important to improve the wearer’s ability to respond to work conditions, but does not eliminate the basic physiological and psychological limits to performance.
